# Supplementary material for: A Cross-Sectional Study of the Satisfaction with, Adherence to, and Perspectives toward COVID-19 Preventive Measures among Public Health Students in Jazan, Saudi Arabia
Source: Int J Environ Res Public Health. 2022 Jan 12;19(2):802. doi: 10.3390/ijerph19020802 (PMC8775640; doi:10.3390/ijerph19020802)
Supplement: Supplementary file 1 [file ijerph-19-00802-s001.zip › ijerph-1502651-supplementary.pdf]

Supplementary Material

File S1: data collection questionnaire, SAP COVID-19 preventive measures

Data Collection Questionnaire

(Satisfaction with, adherence to, and perspectives toward COVID-19 preventive measures)

Dear Public Health Student, Jazan University, Saudi Arabia

This study aims to assess the satisfaction with, adherence to, and perspectives toward COVID-19 preventive measures among public health students in Jazan University, Saudi Arabia. Filling out the questionnaire takes only about seven minutes. Participation in this study is voluntary anonymous, and you can quit this questionnaire at any time. Collected data will be used for research purposes only according to the requirement of the Research Ethics Committee. Your participation is appreciated. Thank you.

For Further Information, you may contact:

Mohammed Almalki
College of Public Health and Tropical Medicine
Jazan University
mjalmalki@jazanu.edu.sa

Thank you very much.

A cross-sectional study of the satisfaction with, adherence to, and perspectives toward COVID-19 preventive measures among public health students in Jazan, Saudi Arabia
Mohammed J. Almalki

1. Are you a public health student at Jazan University, Saudi Arabia?

1 ☐ Yes (please continue to the next question)
2 ☐ No (you may stop here, thank you)

2. Do you agree to participate in this study?

1 ☐ Yes (please start the questionnaire in the next section)
2 ☐ No (you may stop here, thank you)

Answering all questions will enable us to analyze the data of this questionnaire. Please ensure your full participation. Thank you.

PART ONE: Background Information

3. Your gender?

1 ☐ Male
2 ☐ Female

4. Your age? (________________________)

5. Discipline of study?

1 ☐ Epidemiology
2 ☐ Health education & promotion
3 ☐ Health informatics

6. Educational level?

1 ☐ Year 2 students (Levels 3 & 4)
2 ☐ Year 3 students (Levels 5 & 6)
3 ☐ Final year students (Levels 7 & 8)

7. Have you contracted COVID-19?

1 ☐ Yes
2 ☐ No

PART Two: Perspective of organization and dealing, perspective of preventive measures, personal adherence, and general satisfaction with the COVID-19 preventive measures

(Responding to the following questions, please consider your college performance during the current Mid-Term Exam.)

8. Participants' perspective of organization and dealing:

	Strongly Agree	Agree	Neutral	Disagree	Strongly Disagree
**There are health measures to screen people for COVID-19 symptoms before entering the college facilities.**	☐	☐	☐	☐	☐
**Students are received in an organized manner.**	☐	☐	☐	☐	☐
**If there is a problem or concern related to COVID-19, I know exactly how to act and to whom within the college I can refer for support.**	☐	☐	☐	☐	☐

9. Participants' perspective of the COVID-19 preventive measures implemented by the college:

	Strongly Agree	Agree	Neutral	Disagree	Strongly Disagree
**Employees use protective measures such as face masks.**	☐	☐	☐	☐	☐
**Students use protective measures such as face masks.**	☐	☐	☐	☐	☐
**Everyone's temperature is checked immediately before entering the buildings.**	☐	☐	☐	☐	☐
**Everyone is required to use hand sanitizer and face masks before entering the buildings.**	☐	☐	☐	☐	☐
**The safe distancing (2 meters) between people is observed during the screening process and inside the campus.**	☐	☐	☐	☐	☐
**There is a designated location to isolate suspected COVID-19.**	☐	☐	☐	☐	☐
**The college provides personal protective equipment for students and others, as needed.**	☐	☐	☐	☐	☐
**I have received the information and instructions related to COVID-19 preventive measures from the college.**	☐	☐	☐	☐	☐

10. What are the best COVID-19 health education materials provided by the college?

____
____
____
____

11. Personal adherence to the COVID-19 preventive measures on campus:

	Strongly Agree	Agree	Neutral	Disagree	Strongly Disagree
**I wear personal protective equipment such as a face mask.**	☐	☐	☐	☐	☐
**I wash/sanitize my hands as recommended.**	☐	☐	☐	☐	☐
**I adhere to the social distancing measures.**	☐	☐	☐	☐	☐

12. In your opinion, what are the main reasons that prevent students from adhering to the COVID-19 preventive measures on campus?

____
____
____
____

13. General satisfaction with the COVID-19 preventive measures:

	Strongly Agree	Agree	Neutral	Disagree	Strongly Disagree
**I am generally satisfied with the COVID-19 preventive measures implemented in the college during the mid-term exams.**	☐	☐	☐	☐	☐

14. Please list the three most important reasons for your satisfaction/dissatisfaction with the COVID-19 preventive measures implemented in the college.

____
____
____
____

15. In the space below, please write any additional notes or information that you believe will enhance the study results.

____
____
____
____

THANK YOU FOR COMPLETING THIS SURVEY.

أستبانة جمع البيانات

(الرضا عن التدابير الوقائية لكوفيد-19 والالتزام بها ووجهات النظر تجاهها)

عزيزي طالب كلية الصحة العامة بجامعة جازان، المملكة العربية السعودية

تهدف هذه الدراسة إلى تقييم الرضا عن التدابير الوقائية لكوفيد-19 ومدى الالتزام بها ووجهات النظر تجاهها بين طلاب الصحة العامة في جامعة جازان، المملكة العربية السعودية. يستغرق ملء الاستبانة حوالي سبع دقائق فقط. المشاركة في هذه الدراسة طوعية ولا تتطلب معلومات شخصية، ويمكنك التوقف عن ملء هذه الاستبانة في أي وقت. ستستخدم البيانات التي يتم جمعها لأغراض البحث فقط وفقًا لمتطلبات لجنة أخلاقيات البحث. مشاركتك محل التقدير، شكرًا لك.

لمزيد من المعلومات، يمكنك الاتصال بـ:

محمد المالكي
كلية الصحة العامة وطب المناطق الحارة
جامعة جازان
mjalmalki@jazanu.edu.sa

شكرًا جزيلًا.

دراسة مسحية مقطعية حول الرضا عن التدابير الوقائية لـكوفيد-19 والالتزام بها ووجهات النظر تجاهها بين طلاب الصحة العامة في جازان ، المملكة العربية السعودية

1. هل أنت طالب صحة عامة بجامعة جازان بالمملكة العربية السعودية؟

1 ☐ نعم (من فضلك انتقل للسؤال التالي)

2 ☐ لا (بإمكانك التوقف هنا، شكرًا لك)

2. هل توافق على المشاركة في هذه الدراسة؟

1 ☐ نعم (من فضلك ابدأ الاستبانة من السؤال التالي)

2 ☐ لا (بإمكانك التوقف هنا، شكرًا لك)

الإجابة على كافة الأسئلة سيمكننا من التحليل الجيد لبيانات هذه الاستبانة. نأمل الإجابة على جميع الأسئلة. شكرًا لك.

الجزء الأول: معلومات أساسية

3. جنسك؟

1 ☐ ذكر

2 ☐ أنثى

4. عمرك؟ (______________________)

5. تخصصك الدراسي؟

1 ☐ الوبائيات

2 ☐ التثقيف والتعزيز الصحي

3 ☐ المعلوماتية الصحية

6. مستواك الدراسي في الكلية؟

1 ☐ السنة الدراسية الثانية (المستويات 3 و 4)

2 ☐ السنة الدراسية الثالثة (المستويات 5 و 6)

3 ☐ السنة الدراسية الأخيرة (المستويات 7 و 8)

7. هل أصبت بكوفيد-19؟

1 ☐ نعم

2 ☐ لا

الجزء الثاني: وجهة نظر المشاركين تجاه التنظيم والتعامل، وجهة نظر المشاركين تجاه الإجراءات الوقائية المطبقة، الالتزام الشخصي بالإجراءات الوقائية، مستوى الرضا العام عن التدابير الوقائية لكوفيد-19

(عند الإجابة على الخيارات فكر في أداء الكلية التي تدرس بها خلال امتحانات منتصف الفصل الدراسي الحالي.)

8. وجهة نظر المشاركين تجاه التنظيم والتعامل:

	موافق بشدة	موافق	محايد	غير موافق	غير موافق بشدة
**توجد إجراءات صحية للكشف عن أعراض كورونا المستجد (كوفيد-19) عند الدخول إلى مرافق الكلية.**	☐	☐	☐	☐	☐
**يتم استقبال الطلاب بطريقة منظمة.**	☐	☐	☐	☐	☐
**عند وجود مشكلة أو مخاوف تتعلق بـكوفيد-19، أعرف تمامًا كيف أتصرف وبمن أتواصل داخل الكلية للحصول على الدعم.**	☐	☐	☐	☐	☐

9. وجهة نظر المشاركين تجاه الإجراءات الوقائية من كوفيد-19 المطبقة في الكلية:

	موافق بشدة	موافق	محايد	غير موافق	غير موافق بشدة
**يتقيد الموظفون بتدابير الوقاية مثل لبس الكمامات.**	☐	☐	☐	☐	☐
**يتقيد الطلاب بتدابير الوقاية مثل لبس الكمامات.**	☐	☐	☐	☐	☐
**يتم فحص حرارة الجميع بشكل فوري قبل دخول مرافق الكلية.**	☐	☐	☐	☐	☐
**يطلب من الجميع تطهير اليدين بالمطهرات الكحولية ولبس الكمامات قبل دخول الكلية.**	☐	☐	☐	☐	☐
**تطبق المسافة الآمنة (2 متر) بين الأشخاص خلال الفرز وداخل مرافق الكلية.**	☐	☐	☐	☐	☐
**يوجد مكان مخصص لعزل الحالات المشتبه في إصابتها بكورونا المستجد (كوفيد-19).**	☐	☐	☐	☐	☐
**الكلية توفر وسائل الحماية الشخصية للطلاب وغيرهم، حسب الحاجة.**	☐	☐	☐	☐	☐
**تلقيت المعلومات والتعليمات المتعلقة بالوقاية من كوفيد-19 من قبل الكلية.**	☐	☐	☐	☐	☐

10. ما هي أفضل المواد التوعوية التي قدمتها الكلية بخصوص كوفيد-19؟

____
____
____
____

11. الالتزام الشخصي بالإجراءات الوقائية من كوفيد-19 داخل مرافق الكلية:

	موافق بشدة	موافق	محايد	غير موافق	غير موافق بشدة
**أرتدي أدوات الوقاية الشخصية مثل لبس الكمام.**	☐	☐	☐	☐	☐
**أغسل اليدين / أعقم اليدين بالمطهرات الكحولية وفق التوصيات.**	☐	☐	☐	☐	☐
**أتقيد بإجراءات التباعد الاجتماعي.**	☐	☐	☐	☐	☐

12. من وجهة نظركم، ما هي أهم أسباب عدم التزام الطلاب بالإجراءات الوقائية من كوفيد-19 داخل مرافق الكلية؟

____
____
____
____

13. مستوى الرضا العام عن التدابير الوقائية لكوفيد-19:

	موافق بشدة	موافق	محايد	غير موافق	غير موافق بشدة
**أنا راضٍ بشكل عام عن التدابير الوقائية لمواجهة كوفيد-19 المطبقة في الكلية خلال امتحانات منتصف الفصل الدراسي.**	☐	☐	☐	☐	☐

14. من فضلك اذكر أهم ثلاث مبررات لرضاك / عدم رضاك عن الإجراءات الوقائية المطبقة في الكلية.

____
____
____
____

15. في المساحة أدناه، فضلاً أكتب أي ملاحظات أو معلومات إضافية تعتقد أنها ستعزز من نتائج الدراسة.

____
____
____
____

شكرًا لمشاركتك في هذه الاستبانة
